# Extraction of carbon powder from pyrolysis of low-density polyethylene plastics and its application in composite laminates

**DOI:** 10.1016/j.mex.2025.103449

**Published:** 2025-06-19

**Authors:** Kadambari C S Vyasa Krishnaji, Veeresh Kumar GB, Santosh Kumar Sahu, Mohammed Aman

**Affiliations:** a Mechanical Engineering, National Institute of Technology - Andhra Pradesh, Tadepalligudem, Andhra Pradesh 534101, India; b Mechanical Engineering, Sri Vasavi Engineering College, Tadepalligudem, Andhra Pradesh 534101, India; c School of Mechanical Engineering, VIT-AP University, Besides A.P. Secretariat, Amaravati 522237, Andhra Pradesh, India; d Department of Industrial Engineering, College of Engineering, University of Business and Technology, Jeddah 21448, Saudi Arabia

**Keywords:** LDPE, Pyrolysis, Carbon char, Epoxy-based composites, Mechanical properties, The research discusses the extraction of carbon powder from pyrolysis of low-density polyethylene (LDPE) plastics and its application in composite laminates

## Abstract

The escalating environmental concerns associated with plastic waste, particularly Low-Density Polyethylene (LDPE), have spurred research into sustainable recycling strategies. Pyrolysis has been developed as a viable technique for transforming LDPE into appreciated by-products, including carbon powder, which holds potential for advanced material applications. This study investigates the extraction of carbon powder from LDPE via pyrolysis and its subsequent utilization in composite laminates.

• Araldite LY 556 and Aradur HY 951 epoxy resins are used to create the laminates, and carbon and kevlar fiber reinforcement are added in different weight fractions of 0 to 30 % in the intervals of 10 % of carbon char generated from LDPE.
• Tensile strength (ASTM D638), flexural strength (ASTM D790), X-ray diffraction (XRD), Fourier Transform Infrared Spectroscopy (FT-IR) and scanning electron microscopy (SEM) are used to do a thorough assessment of mechanical and structural features.
• Incorporation of LDPE-derived carbon char significantly enhanced mechanical properties of epoxy-based laminates. At 30 wt % char, tensile strength increased by 75 % in carbon and 129 % in Kevlar composites, while flexural strength improved by 94 % and 196 %, respectively. SEM, XRD, and FTIR analyses confirmed improved interfacial adhesion, structural integrity, and filler stability, demonstrating char’s effectiveness as a sustainable reinforcement.

## Graphical abstract

**Figure:**
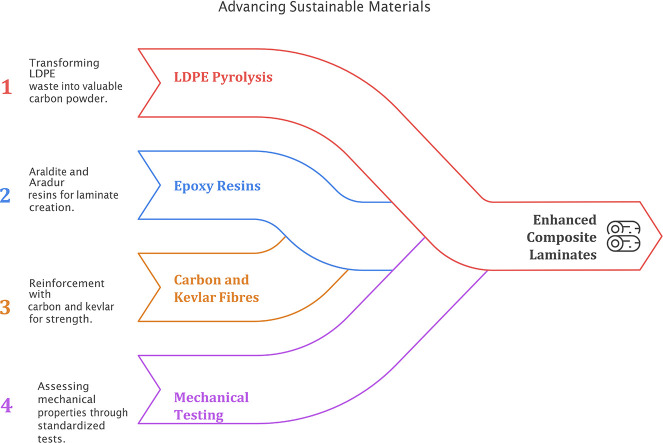

Specifications table

**Table:** 

Subject area:	Materials Science
More specific subject area:	Polymer Composites by Recycling LDPE plastics.
Name of your method:	The research discusses the extraction of carbon powder from pyrolysis of low-density polyethylene (LDPE) plastics and its application in composite laminates
Name and reference of original method:	None
Resource availability:	None

## Background

The escalation of plastic waste, particularly Low-Density Polyethylene (LDPE), demands green recycling technologies. Pyrolysis, a thermally induced degradation process, converts LDPE into carbon-based products, which increase composite laminates. Carbon-reinforced composites possess improved mechanical properties and find applications in aerospace, automotive, and marine sectors. The synthesis of LDPE-based carbon char minimizes waste, conserves virgin material, and promotes circular economy. The widespread availability of LDPE waste ensures cheap mass production, with high-wear applications like gears and bearings exploiting its hardness [1,2]. Schade et al. [3] elaborated on recycling technology, stating mechanical recycling leads to downcycling and chemical recycling struggles to be energy efficient as well as separate. Pyrolysis and gasification, while primarily fuel-producing, are improved by catalytic and plasma-assisted processes. Pyrolysis, being scalable to large scale, produces high-value Carbon Nano Materials (CNMs) through Flash Joule Heating (FJH), Chemical Vapor Deposition (CVD), and thermal decomposition [4]. While FJH produces graphene at ₹85–100/kg instantly, scalability is limited by infrastructure constraints. CVD offers pure products, but they are costly and complicated. Yaqoob et al. [1] explored the upcycling of plastic wastes into carbon nanotubes (CNTs), graphene, and porous carbon for energy storage applications. Pyrolysis and catalytic degradation are highly regulated processes, while transparent waste collection policies ensure maximum recycling efficiency. Blanchard et al. [2], demonstrated that LDPE, Polyethylene Terephthalate (PET) and polyolefins can be converted into activated carbon (AC) with greater than 2000 m²/g surface area by KOH activation. The method improves the adsorption of contaminants and presents a promising alternative to coal- and biomass-based AC but must be optimized for commercial use. Shah et al. [5] have pointed out pyrolysis' efficiency relative to gasification for plastic waste conversion, depending on reactor design, temperature, pressure, and catalyst. The processes conserve chemical energy more than incineration and reduce landfill waste.

Peti et al. [6] have suggested enzymatic degradation, depolymerization, and pyrolysis as replacements for mechanical recycling, with AI-assisted sorting increasing sustainability. Achilias et al. [7] revealed that pyrolysis catalytic produces hydrocarbons that can be recycled in petrochemical processes. Sivagami et al. [8], revealed that ZSM-5 zeolite LDPE pyrolysis gave 70 wt. % oil and 14 wt. % char with a calorific value of 41 kJ/g, comparable to diesel. Rajan et al. [9] achieved a 67.3 % yield of oil employing ZnO as a catalyst, with efficiency at low temperatures. Wijayanti et al. [10] reported LDPE pyrolysis at 350 °C to produce 90 % fuel oil of commercial-grade quality, which can be further enhanced by refining and additives. Dallaev et al. [11] highlighted epoxy resins' superior mechanical and dielectric properties for applications in high-performance products. Qin et al. [12] identified Hybrid Glass-Carbon FRP (HFRP) and Carbon Fiber Reinforced Polymer (CFRP) composites with enhanced tensile strength. Pyrolysis carbons have high carbon composition (95 %) with minimal impurities [13]. Solid carbon char, produced by five-hour slow pyrolysis, is added to epoxy matrices to form composite laminates. These composites improve structural integrity and electrical conductivity, expanding their use in sustainable materials [[14], [15], [16], [17], [18], [19], [20]] .

## Method details

### Composite laminate production

**Fig. 1:**
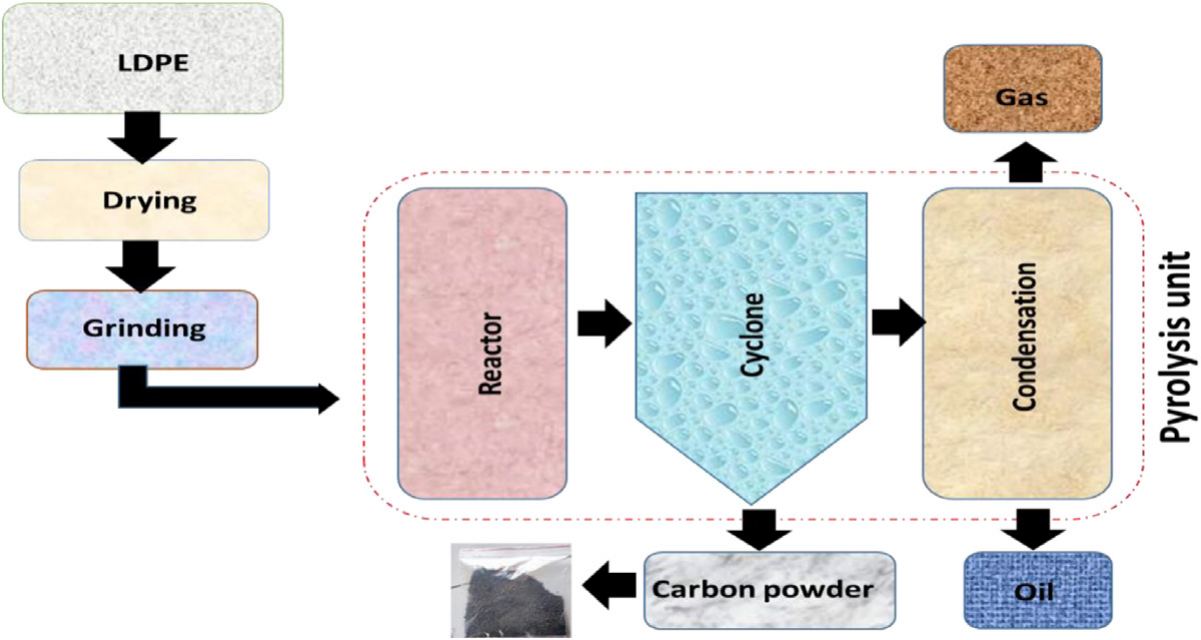
LDPE char derived from pyrolysis.

**Fig. 2:**
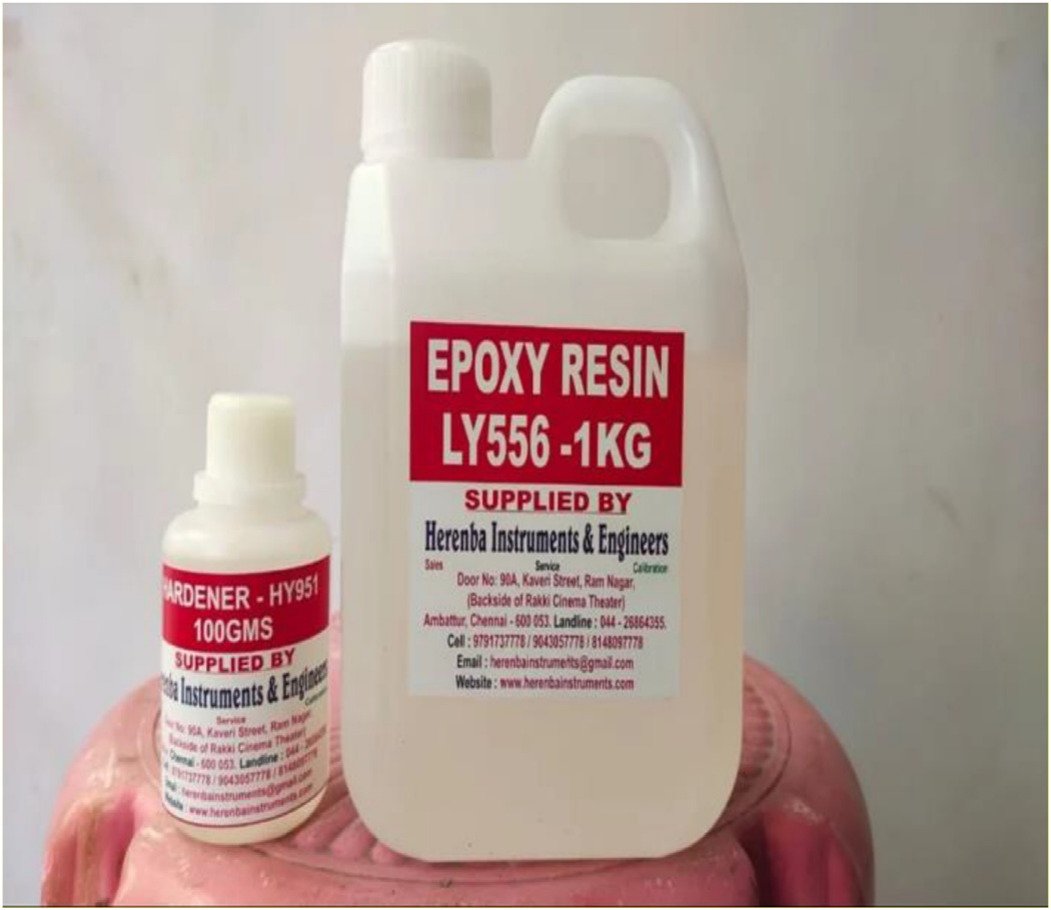
Epoxy LY556 and Hardener HY951.

The extracted carbon powder from LDPE pyrolysis (Fig. 1) can be incorporated into composite laminates using epoxy resin systems such as Araldite LY 556 and Aradur HY 951 hardener, shown in Fig. 2. Epoxy systems are well known to be a significant composite material for low-temperature applications, typically <200°F (93 °C). They have a variety of desirable properties that make them extremely useful for industries and uses. One of the main strengths of epoxy systems is their excellent chemical resistance. They resist various chemicals, such as acids, alkalis, solvents, and fuels. This property is especially beneficial when the composite material can be exposed to corrosive materials or environments. In addition, epoxy systems have better adhesion to fibres, which makes them well-suited for application in composite materials.

The composites of epoxy can have superior mechanical properties and high strength-to-weight ratios with reinforcement from reinforcement fibres, e.g., carbon or glass fibres. The strong intermolecular interactions of the fibres and epoxy matrix provide the toughness and load capacity of the composite. Dimension stability is also a commendable feature of epoxy systems. The composites exhibit stability and precise dimensionality control as low shrinkers upon cure. It is a distinctive attribute of technology, such as precision engineering or aerospace applications, where high accuracy and dimensional tolerance are required. Further, epoxy systems can be tailored by using various fillers and additives to meet application requirements [12].

### Materials used

**Fig. 3:**
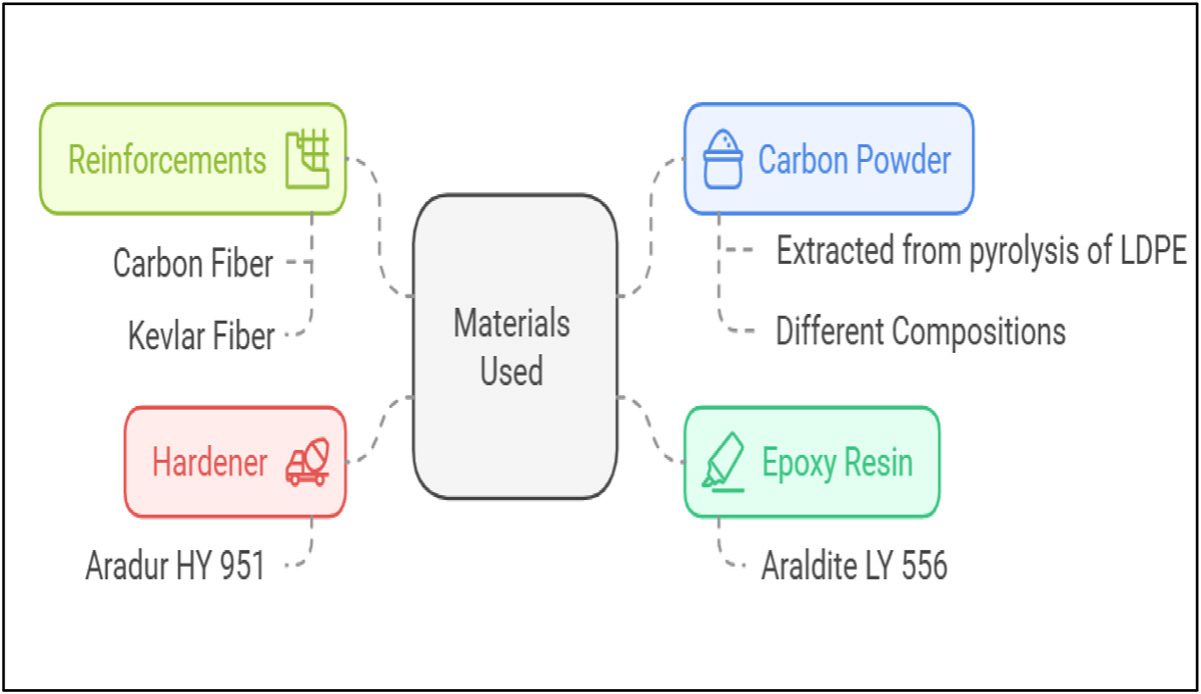
Materials used for laminate preparation.

The complete arrangement of the materials used during composite laminate preparation is illustrated in Fig. 3, with greater emphasis on the most significant contributors: epoxy resin, reinforcement, hardener, and carbon powder. Araldite LY 556, a high-strength epoxy resin, finds predominant application as a matrix material due to its superior mechanical properties and resistance against chemical compounds or attacks. Aradur HY 951 as a curing aid for improving cross-link density of the LY 556 epoxy matrix and structural stiffness of the composite enables curing. The reinforcing material is Carbon and Kevlar fibres with high mechanical strength, modulus, and resistivity and are applied in high-performance composite parts. In addition, to further improve the material's electrical conductivity, heat stability, and overall properties, pyrolysed LDPE is added to the composite system with different compositions of carbon powder. These materials work together to create high-value, multipurpose laminates appropriate for structural and functional applications, showcasing a strategic approach to sustainable composite creation using recycled carbon sources.

### Laminate composition

**Fig. 4:**
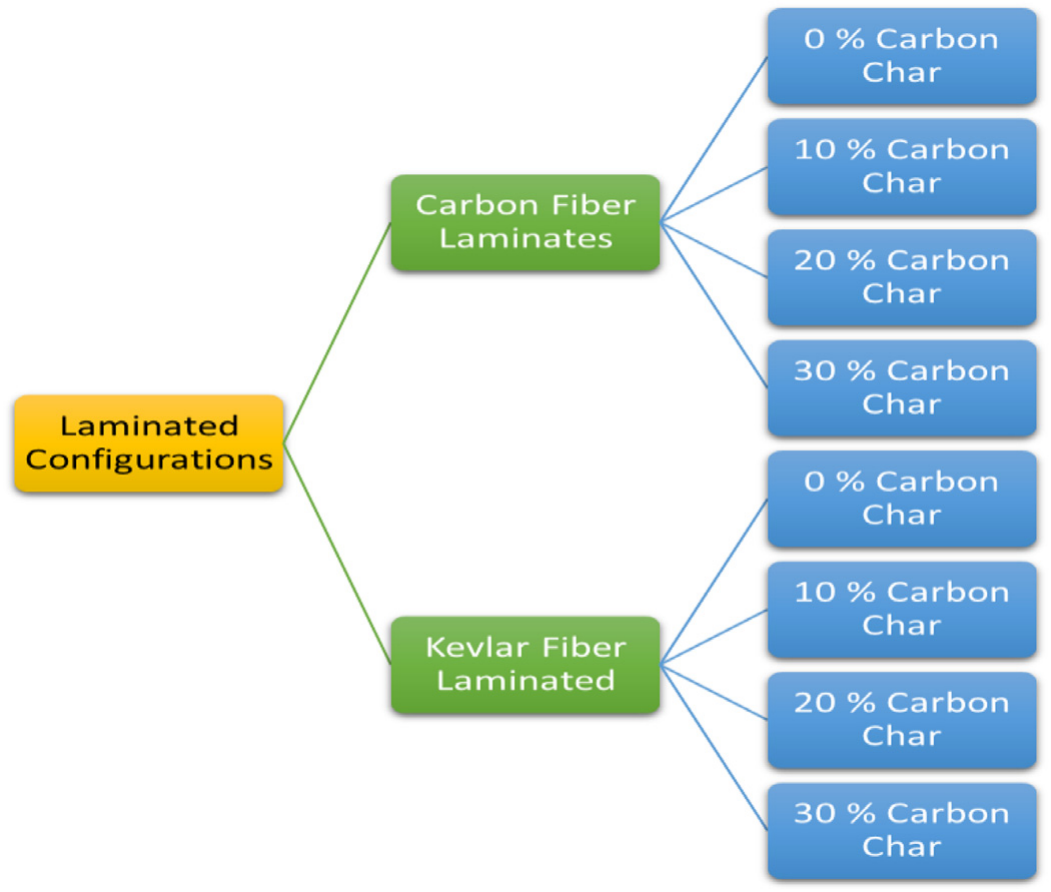
Laminate composition configurations.

Fig. 4 shows the eight different laminate configurations produced based on varying compositions of carbon char.

### Production process

The LY 556 epoxy resin is combined with the HY951 hardener in a precise weight ratio 100:10 to ensure optimal cross-linking and mechanical integrity. Preheating the epoxy mixture enhances its flow characteristics, facilitating better impregnation of the reinforcing fibres. To achieve uniform dispersion, carbon char is combined into the LY 556 epoxy matrix at predetermined weight fractions of 0 %, 10 %, 20 %, and 30 %, followed by thorough mixing. The homogenised resin-char mixture is then infused onto layers of carbon or kevlar fibres, which are strategically arranged within a mould. A vacuum bagging technique enhances fibre wetting and eliminates entrapped air, producing high-quality laminates with minimal void content [21,22]. Ultrasonication-assisted curing is integrated into the fabrication process to accelerate the polymerisation reaction and enhance the mechanical performance of the composite. This is achieved using an ultrasonic homogeniser (VCX 750, Sonics and Materials Inc.), which operates at a 20 kHz frequency and a 750 W power output, facilitating improved dispersion of carbon char particles and reducing viscosity for superior matrix-fiber adhesion.

**Fig. 5:**
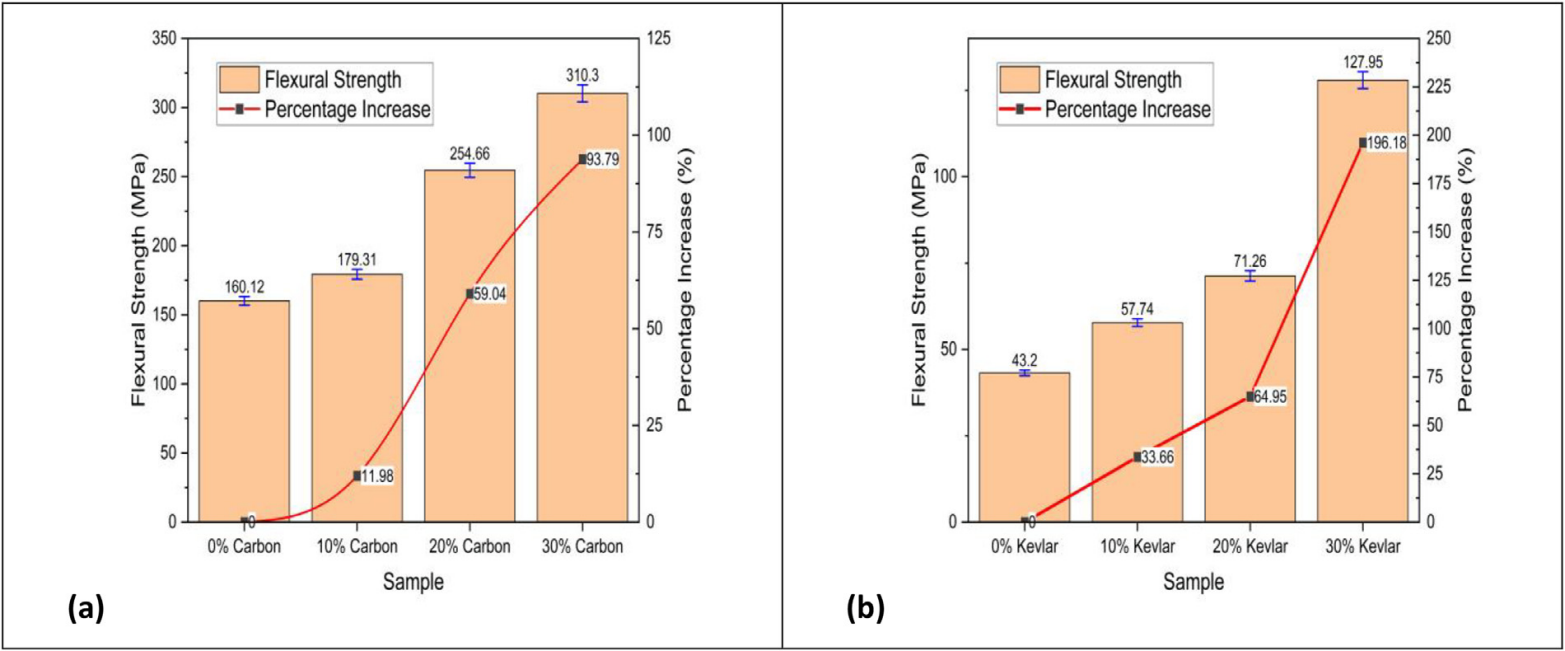
Tensile strength vs Carbon content of (a) carbon fibre laminates and (b) kevlar fibre laminates.

**Fig. 6:**
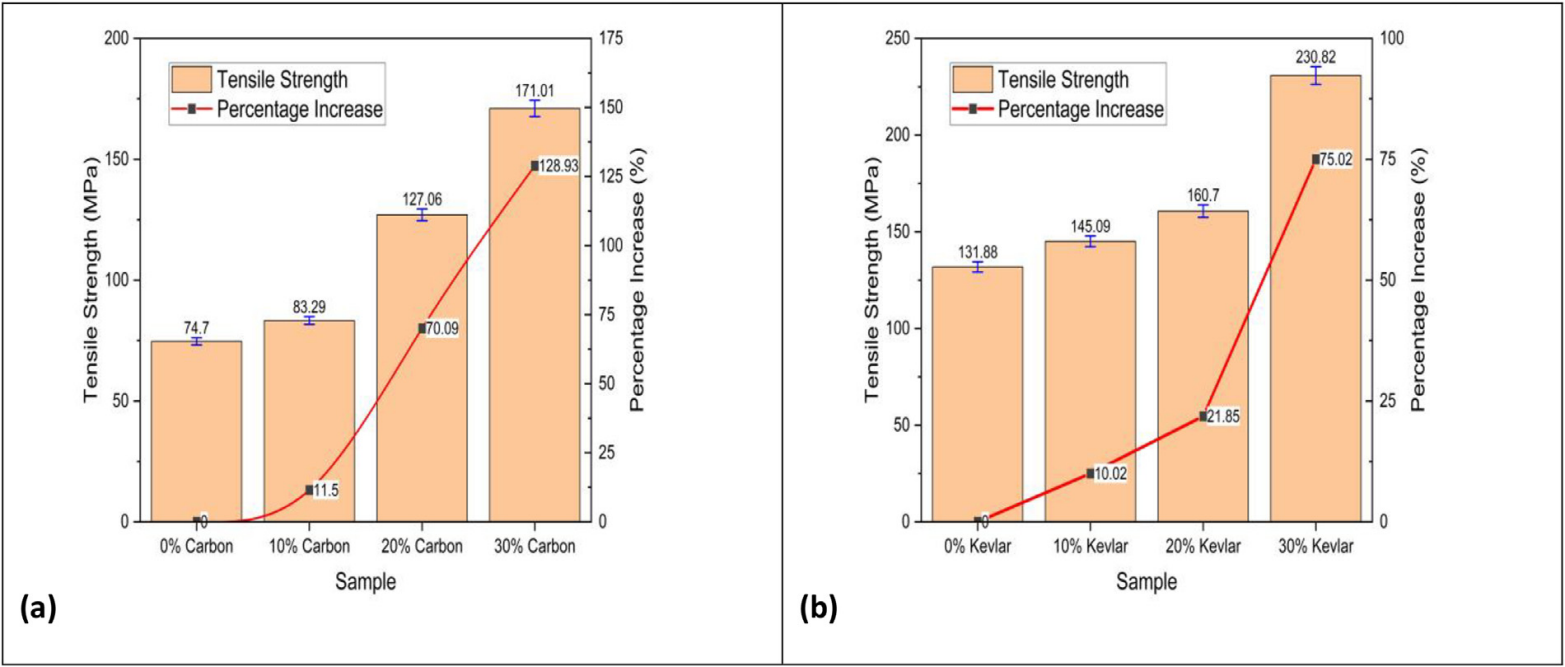
Flexural strength vs Carbon content of (a) carbon fibre laminates and (b) kevlar fibre laminates.
• The higher flexural strength observed in kevlar fiber-reinforced composites compared to carbon fiber laminates at elevated carbon char contents is primarily due to key structural and interfacial differences. Kevlar fibers contain polar amide groups capable of forming strong hydrogen bonds and polar interactions with oxygen-containing functional groups on the char surface, leading to superior interfacial adhesion and more effective stress transfer. In contrast, carbon fibers are chemically inert and graphitic, exhibiting weaker interactions with the char and, thus, less efficient load transfer. Additionally, the rough surface morphology of kevlar fibers enhances mechanical interlocking with the matrix and char particles—particularly important at higher filler loadings—while the smoother surface of carbon fibers limits this benefit. Kevlar’s ductility and flexibility also help dissipate stress concentrations caused by filler agglomeration or porosity, whereas the rigidity of carbon fibers increases the likelihood of crack initiation under flexural loads. Kevlar also exhibits better wetting with polar matrices modified by oxidized char, strengthening the fiber–matrix–filler interface. Carbon fibers, by contrast, do not significantly improve matrix wetting. Collectively, these factors—stronger interfacial bonding, improved stress accommodation, and better mechanical interlocking—contribute to the superior flexural performance of Kevlar composites at high char loadings [28].

• The last curing procedure is carried out in a temperature-controlled environment to attain the best mechanical qualities. To achieve complete polymerisation and reduce residual stresses within the composite structure, the laminates are cured stepwise for six hours at an elevated temperature of 80 °C. This prolonged processing method produces laminates suitable for high-tech applications because they are structurally stable and exhibit excellent mechanical and thermal stability [23].
• **Mechanical Testing, Characterization and Results Analysis**
  To determine the performance of the produced laminates, various mechanical tests are carried out:
  **Tensile Strength and Modulus (ASTM D638):** Under this test, the tensile or strain that a material can absorb before breaking is measured. One key aspect in analysing the mechanical properties of fibre-reinforced composites under load conditions is their tensile strength. Fig. 5 shows how the tensile strengths of carbon fibre laminates (Fig. 5a) and kevlar fibre laminates (Fig. 5b) change with added carbon content.
• The experimental outcomes indicate a noteworthy enrichment in tensile strength with the amalgamation of carbon content. For carbon fibre laminates, an initial increase of 10 % carbon results in an approximate 10 % improvement in tensile strength, with a more pronounced 44 % increase observed at 30 % carbon content.
• Similarly, for kevlar fibre laminates, the tensile strength exhibits a 12 % increase at 10 % carbon content, followed by a substantial 53 % rise at 20 % carbon, ultimately reaching a 35 % increase at 30 % carbon content. This trend suggests that the carbon reinforcement contributes to the upgraded load-bearing capability by enhancing interfacial bonding and matrix-fibre load transfer efficiency. Compared to kevlar-based composites, the higher tensile strength observed in carbon fibre laminates can be attributed to carbon fibre's superior stiffness and inherent strength. These findings are crucial for the development of high-performance composite materials in aerospace, automotive, and structural applications, where optimising mechanical properties is essential for lightweight and durable material solutions [12].
• **Flexural Strength (ASTM D790):** A three-point bending examination assesses how well a material can resist deformation under applied load. Flexural strength is critical in determining fibre-reinforced composites' load-bearing capability and structural integrity. Fig. 6 illustrates the variation in flexural strength with increasing carbon content for both carbon fibre laminates (Fig. 6a) and kevlar fibre laminates (Fig. 6b). The results reveal a notable enhancement in flexural strength with increasing carbon content, suggesting improved resistance to bending deformation. For carbon fibre laminates, an initial 10 % carbon addition flexural strength was enhanced by 12 %, with a more pronounced 42 % and 22 % increase observed at 20 % and 30 % carbon content, respectively. In contrast, kevlar fibre laminates exhibit a substantial 34 % improvement at 10 % carbon content, followed by a 23 % increase at 20 % and a remarkable 80 % increase at 30 %. These findings indicate that carbon reinforcement improves the composites' flexural strength, with a more pronounced effect observed in kevlar-based laminates at higher carbon content. This can be attributed to the improved fibre-matrix adhesion, enhanced stress transfer mechanisms, and superior mechanical properties of carbon fibres. These perceptions are decisive for the design of lighter-weight, high-performance composite structures utilised in aerospace, automotive, and defence applications where flexural performance is paramount [[24], [25], [26], [27]].

### Scanning electron microscopy

**Fig. 7:**
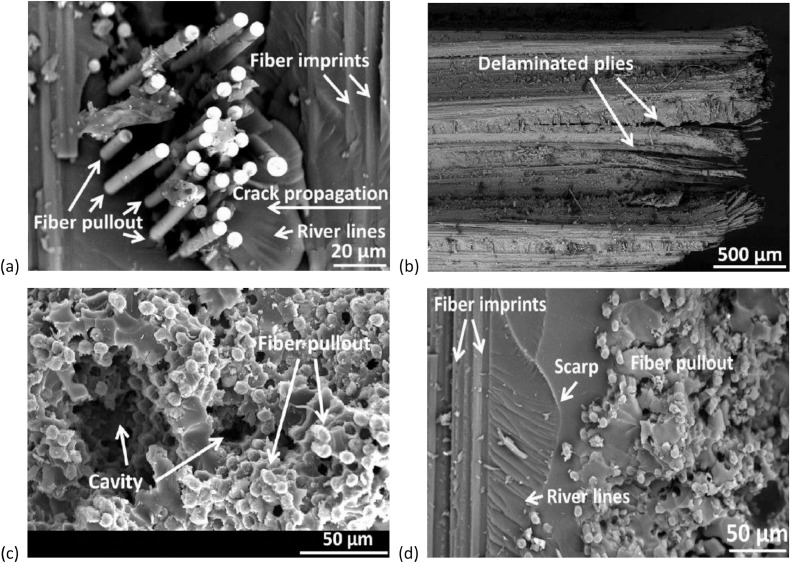
SEM images of laminates after tensile strength and flexural strength tests are conducted on the laminates.(a) Fiber-Matrix Interfacial Failure Indicating Fiber Pullout, Crack Propagation, and River Line Formation in Fiber-Reinforced Epoxy Composite. (b) SEM Image Showing Interlaminar Delamination and Ply Separation in Fiber-Reinforced Epoxy Composite. (c) Sem Image Revealing Fiber Pullout and Cavities Indicative of Weak Interfacial Adhesion in Fractured Composite. (d) Fracture Surface Morphology Showing Fiber Pullout, Imprints, and River Lines Indicating Brittle Failure in Composite Interface.

The SEM analysis provides very important insights into the surface morphology and fiber-matrix adhesion within fiber-reinforced epoxy composites. The SEM pictures presented in Fig. 7 reveals the fracture mechanisms of carbon and kevlar fiber-reinforced epoxy laminates incorporating varying weight fractions (0 %, 10 %, 20 %, and 30 %) of carbon powder derived from the pyrolysis of LDPE. These microstructural observations illustrate the influence of carbon char as a filler material on interfacial bonding, failure modes, and overall mechanical integrity [23,29,30]. The presence of fiber pullout and fiber imprints suggests inadequate adhesion between the epoxy matrix and reinforcing fibers, indicating that at certain filler concentrations, the compatibility between the resin and fiber reinforcements may be compromised. Additionally, the observed river lines in the SEM micrographs indicate the direction of crack propagation within the matrix, with lower filler concentrations exhibiting brittle failure characterized by cleavage fractures. As the filler content increases, the fracture behavior transitions from a purely brittle mode to a more complex failure mechanism involving micro-crack deflection and energy dissipation [31]. Furthermore, delaminated plies in laminates with higher filler concentrations highlight a reduction in interlaminar strength, likely due to void formation and non-uniform filler dispersion. This suggests that excessive carbon char content can act as stress concentration sites, leading to premature delamination under mechanical loading [32]. The impact of carbon char on the composite's porosity is further highlighted by the existence of cavities and matrix voids; larger filler loadings (20 % and 30 %) result in increased porosity, which may negatively impact the material's flexural and tensile properties [33]. These voids, which further enhance the mechanical performance of the composite, most likely result from insufficient mixing, air entrapment, or resin shrinkage after curing. Furthermore, the SEM pictures' rough fractured surfaces and scarp formation imply that the dispersion of carbon powder made from pyrolyzed LDPE leads to microstructural heterogeneity, which influences wear resistance and crack initiation [33].

### X-ray diffraction (XRD)

The phase composition and microstructural properties of polymer-reinforced fiber composites have a significant impact on their performance and structural integrity. XRD serves as a fundamental tool for elucidating the crystalline and amorphous phases within these materials, offering insights into their compatibility, dispersion, and overall reinforcement effectiveness. In recent years, carbonaceous additives, such as carbon powder, have been increasingly combined into epoxy matrices to improve mechanical strength, electrical conductivity, and thermal stability. Nonetheless, degree of structural order and the interaction between the epoxy matrix, reinforcing fibers, and carbon fillers remain critical factors governing composite performance. This study employs XRD analysis to investigate the crystallographic features of carbon and kevlar fiber laminates with varying carbon powder content, aiming to elucidate their structural evolution and potential implications for advanced engineering applications.

### XRD analysis of carbon fiber laminates

**Fig. 8:**
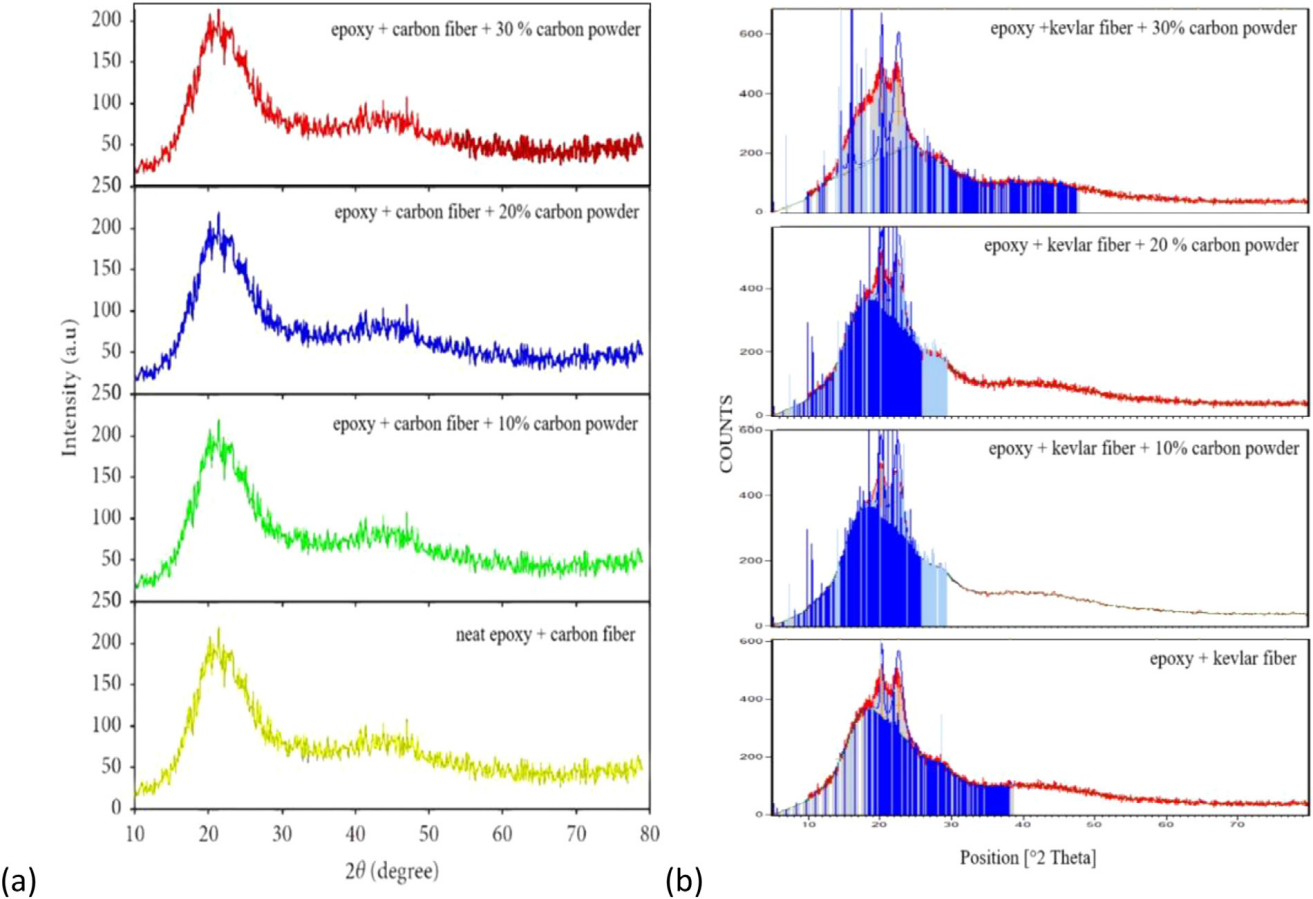
XRD Analysis of (a) Carbon fiber laminates and (b) Kevlar fiber laminates.

The most prominent peak appears at approximately 20–22 degrees for all samples, can be seen in Fig. 8, which is typical for carbon-based materials and represents the (002) graphitic plane. The broad nature of the main peak indicates partial crystallinity in the matrix and presence of amorphous regions. As the carbon powder content increases from 10 % to 30 %, several changes are observed such as peak intensities slightly increase, background noise remains relatively consistent and secondary peaks around 40–50 degrees become more pronounced. The consistent peak patterns across different concentrations indicate good compatibility between the epoxy matrix, carbon fibers, and carbon powder, suggesting stable composite formation [34].

### XRD analysis of kevlar fiber laminates

The XRD spectrum highlights the predominance of amorphous epoxy with minimal contribution from semi-crystalline kevlar fibers. The broad peak at ∼20° confirms the material's amorphous nature, which provides toughness and flexibility, while kevlar fibers enhance mechanical performance. This combination makes the composite appropriate for protective and high-performance applications. The absence of changes in the XRD patterns for composites with increasing carbon powder content is primarily due to the amorphous nature of the epoxy matrix and the carbon powder itself. Other factors, such as aggregation, poor dispersion, and instrument sensitivity, also contribute to the absence of detectable deviations [35,36].

### Fourier transform infrared spectroscopy

Understanding the fiber-reinforced polymer composites chemical interactions and structural integrity is of very crucial for optimizing their performance in advanced engineering applications. FT-IR serves as a powerful analytical technique for identifying functional groups and assessing the chemical bonding within composite materials. FTIR analysis offers important information about the curing process, matrix-fiber interactions, and the function of carbon powder as a reinforcing agent in epoxy-based laminates reinforced with carbon and kevlar fibers. Although carbonaceous fillers are frequently used to improve mechanical and thermal qualities, it is still unclear how they affect the chemical structure of the composite. In order to assess the contribution of carbon powder to improving composite stability and performance, this work uses FT-IR spectroscopy to examine the chemical makeup of carbon and kevlar fiber laminates with different carbon powder contents. The results advance our knowledge of material behavior in high-performance applications, where there is a growing need for composites that are strong, lightweight, and thermally stable.

**Fig. 9:**
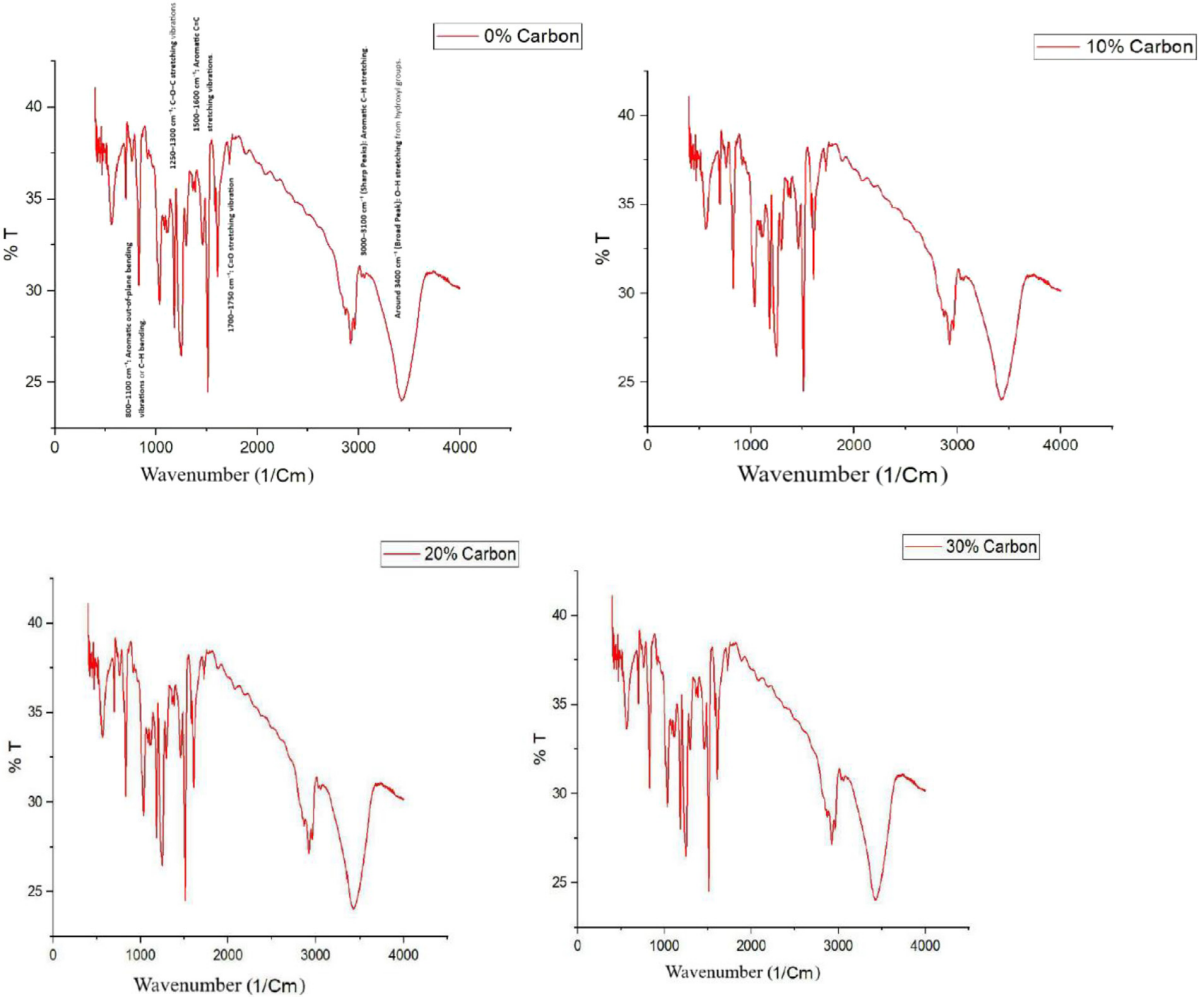
FTIR Analysis of carbon fiber laminates.

**Fig. 10:**
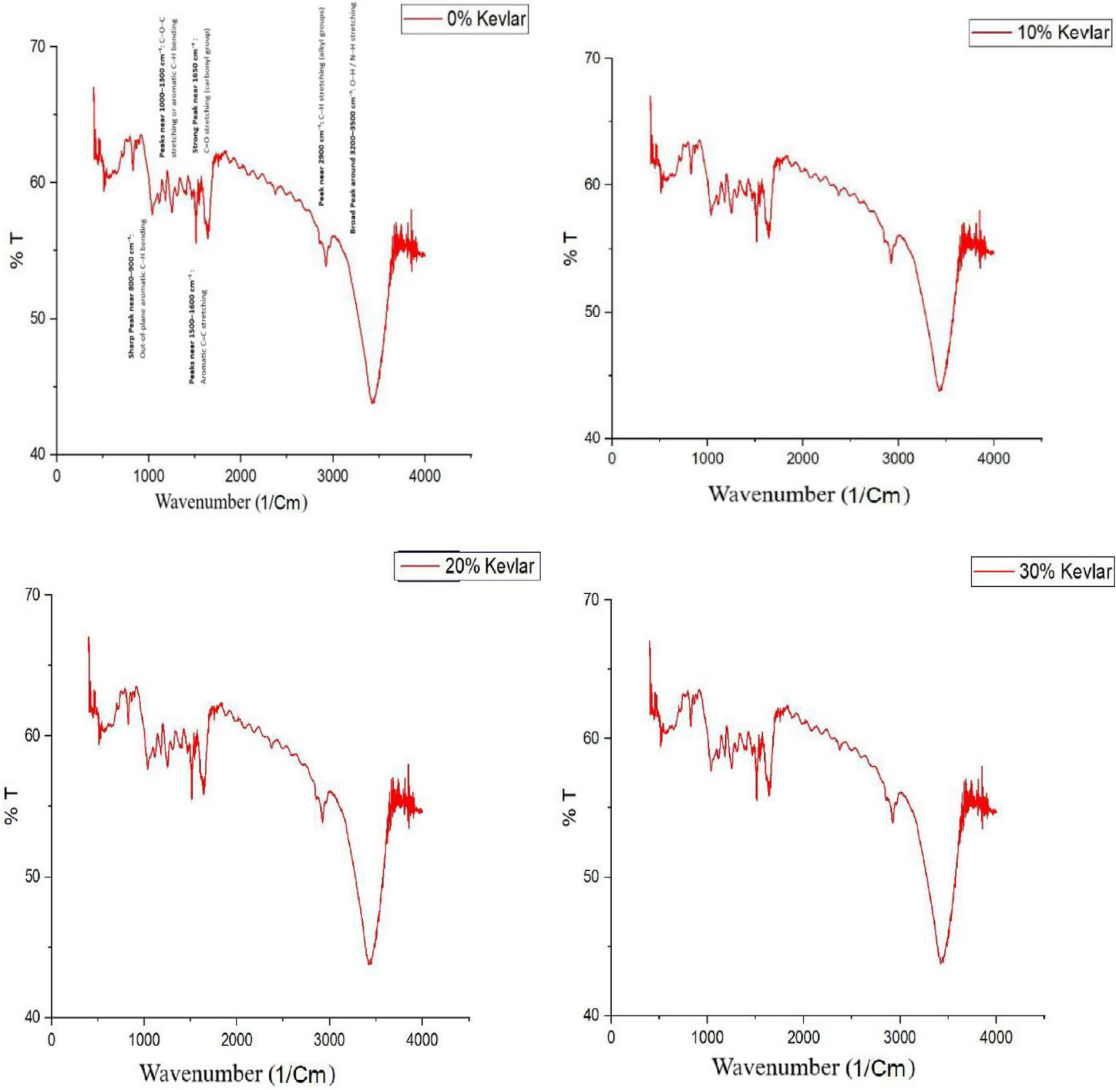
FTIR Analysis of kevlar fiber laminates.

Fig. 9, Fig. 10 of FTIR spectrum show that the epoxy matrix is well cured and has significant carbonyl and aromatic groups. FTIR spectrum of epoxy-kevlar/carbon fiber composite shows intense chemical interactions and a perfectly developed structure, blending the heat resistance and rigidity of the fibers and toughness and flexibility of the epoxy matrix. Instead of participating in any chemical reaction or substantially affecting the functional groups of the composite, the addition of carbon powder is acting as a physical filler. This is enhancing the mechanical properties of the composite by physical reinforcement and not by chemical interaction. The non-alteration of the FTIR spectra with increasing proportion of carbon powder is due to the substitution inertness of the carbon powder. This composite holds a lot of promise for high-performance applications, especially in applications with lightweight, strength, and thermally stable materials [37,38].

### Method validation

• **Tensile Strength and Modulus (ASTM D638):** Generally, an increase in carbon char content leads to improved tensile strength up to an optimal point, beyond which excessive filler may introduce voids or reduce interfacial bonding, resulting in decreased strength. The study demonstrates that LDPE-derived carbon char significantly improves the tensile strength of carbon and kevlar fibre laminates. Carbon fibre laminates exhibit a 75 % increase in tensile strength at 30 % carbon char, rising from 131.88 MPa (0 % carbon char) to 230.82 MPa. In contrast, kevlar fibre laminates show an even more significant improvement of 129 %, increasing from 74.70 MPa (0 %) to 171.01 MPa (30 %). These findings suggest that kevlar-based laminates benefit more from carbon char reinforcement than carbon fibre laminates, likely due to enhanced interfacial bonding and stress distribution. The sharp increase in tensile strength at 30 % carbon char indicates a potential percolation threshold, optimising load transfer and matrix strengthening. While the overall trend is positive, further research is required to explore fracture toughness, wear resistance, and long-term durability. These results confirm the prospective of LDPE-derived carbon char as a justifiable filler, advancing eco-friendly composite materials while addressing plastic waste valorisation for high-performance engineering applications [39].
• **Flexural Strength (ASTM D790):** Similar trends are observed in flexural strength, where moderate inclusion of carbon char enhances rigidity, while excessive amounts may lead to brittleness due to poor dispersion. The study demonstrates that LDPE-derived carbon char significantly enhances the flexural strength of both carbon fibre and kevlar fibre laminates. In carbon fibre laminates, the flexural strength increased from 160.12 MPa at 0 % carbon char to 310.30 MPa at 30 % carbon char, marking a 94 % improvement. The highest rate of increase was observed between 10 % and 20 % carbon char (42 %), indicating effective reinforcement. In kevlar fibre laminates, the flexural strength rose from 43.20 MPa at 0 % to 127.95 MPa at 30 %, demonstrating a considerable increase - more than double the enhancement in carbon fibre laminates. The highest flexural strength growth occurred at 30 % carbon char (80 % increase from 20 %), suggesting strong interfacial bonding. Kevlar composites benefitted more from carbon char reinforcement than carbon fibre laminates, highlighting its potential for lightweight, high-strength structural applications [40,41].
• **Morphological Insights from SEM:** In summary, the SEM analysis shows that, depending on its content, carbon char produced from LDPE can play the dual roles of reinforcing and possibly weakening. The structural integrity of the composite material is compromised by excessive filler content (30 %), which encourages porosity and delamination, while moderate filler additions (10 %–20 %) seem to improve fracture resistance. To fully realize the potential of carbon powder as a justified reinforcement in fiber-reinforced composites, our findings highlight the necessity of refining filler dispersion tactics and optimizing processing methods [[42], [43], [44], [45]]. Moderate carbon inclusion usually results in better fiber-matrix adhesion, according to SEM images; however, too much filler may cause more voids or poor dispersion. According to the SEM examination, carbon char produced from LDPE affects the porosity, fiber-matrix adhesion, and fracture behavior of carbon and kevlar fiber laminates. While heavy loading (30 %) causes porosity, delamination, and decreased interlaminar strength, moderate filler content (10 %–20 %) improves crack resistance. For fiber-reinforced composites to have the best possible mechanical performance and structural integrity, filler dispersion and processing methods must be optimized [40,46,47].
• **Insights from XRD Analysis:** The XRD analysis confirms that carbon fiber laminates exhibit a pronounced graphitic peak (∼20–22°), with increasing carbon powder content (10 %−30 %) enhancing structural order and reinforcing the composite. In contrast, kevlar fiber laminates retain a highly amorphous nature, showing minimal peak variations. These findings indicate effective reinforcement in carbon fiber composites, while kevlar laminates maintain flexibility, requiring improved filler dispersion for enhanced properties [48,49].
• **Insights from FTIR Analysis:** The FTIR analysis of epoxy-based carbon and kevlar fiber laminates confirms the presence of well-cured epoxy matrices with characteristic aromatic, carbonyl, and hydroxyl functional groups. The spectra reveal that the addition of carbon powder (10 %−30 %) does not induce significant chemical changes, indicating that it primarily acts as a physical filler rather than participating in chemical interactions. While carbon fiber laminates exhibit better compatibility with carbon powder, kevlar laminates show minimal spectral variations, suggesting limited interaction. These conclusions highpoint the composite systems chemical stability, making them appropriate for high-performance applications requiring durable, lighter weight, and thermally stable materials [[50], [51], [52]].
• The observed enhancements in tensile and flexural strength with increasing LDPE-derived carbon char content can be attributed to improved load transfer, enhanced fiber-matrix adhesion, and effective stress distribution facilitated by the filler. At moderate filler levels (10–20 %), the carbon char serves as an efficient reinforcing agent, occupying voids and promoting interfacial bonding between fibers and matrix. In kevlar laminates, the relatively higher percentage improvements suggest that the inherently rougher fiber surface morphology may provide better mechanical interlocking with the char particles. However, beyond the optimal content (∼30 %), excessive filler tends to agglomerate, increasing porosity and leading to stress concentrations, as confirmed by SEM, which compromises the composite's mechanical integrity. These results reflect a typical percolation behavior where a critical filler threshold enhances performance before leading to diminishing returns, thereby underscoring the importance of optimizing filler dispersion and content.
• **Future Outlook on Environmental Sustainability:**
  While the present study successfully demonstrates the reinforcement potential of LDPE pyrolysis char in fiber-reinforced polymer composites, a comprehensive evaluation of the environmental benefits is essential to fully validate its sustainability claims. Future work can incorporate a detailed life cycle assessment (LCA) to compare the environmental impact of using pyrolysis-derived carbon fillers with conventional recycling or disposal methods. Such an analysis would provide valuable insight into energy consumption, emissions, and resource efficiency across the material’s lifecycle. Integrating LCA with mechanical performance evaluation will support the development of truly sustainable composite materials aligned with circular economy principles.

## Limitations

None.

## Ethics statements

Not applicable.

## CRediT authorship contribution statement

**Kadambari C S Vyasa Krishnaji:** Validation, Formal analysis, Investigation, Data curation, Resources, Writing – original draft. **Veeresh Kumar GB:** Conceptualization, Methodology, Writing – review & editing, Supervision, Project administration. **Santosh Kumar Sahu:** Formal analysis, Investigation, Data curation, Visualization, Writing – review & editing. **Mohammed Aman:** Formal analysis, Investigation, Data curation, Visualization, Writing – review & editing.

## Declaration of Competing Interest

The authors declare that they have no known competing financial interests or personal relationships that could have appeared to influence the work reported in this paper.

## Data Availability

No data was used for the research described in the article.
